# Early-life experience and disease exposure affect pathogen avoidance behaviour in macaques

**DOI:** 10.1098/rsos.260011

**Published:** 2026-10-21

**Authors:** Stephan P. Kaufhold, Amanda Goguen, Kyliegh Viano, Federico Rossano

**Affiliations:** ^1^ Department of Cognitive Science, University of California San Diego, La Jolla, CA, USA; ^2^ Born Free USA, Silver Spring, MD, USA

**Keywords:** pathogen avoidance, behavioural immune system, disgust, macaque

## Abstract

Pathogens exert strong selective pressure on group-living primates. In humans, disease defence includes both a biological and behavioural immune system, which includes the emotion of disgust and avoidance behaviours shaped by genetics, learning and culture. While pathogen avoidance is documented in nonhuman primates, little is known about how ecological and developmental factors influence pathogen sensitivity, that is, individual variation in pathogen avoidance. We conducted two studies to examine this in macaques. Our first study tested macaques (*n* = 54) from seven different species and four different rearing backgrounds in a visual food-choice task where food was either placed on a substrate with or without pathogenic cues. Macaques preferred feeding on non-contaminated substrates, with sanctuary-born individuals showing greater avoidance than former pets. Our second study tested sanctuary-born Japanese macaques (*n* = 55) from four groups; three groups had recently experienced an infectious disease outbreak. Macaques from exposed groups showed significantly higher avoidance of contaminated substrates. Together, our findings suggest that early life rearing experience and recent disease exposure shape pathogen sensitivity in macaques, demonstrating a flexible behavioural immune system in Old World monkeys. These findings suggest that flexible, context-sensitive pathogen avoidance is present in Old World monkeys, providing the behavioural foundations from which human disgust may have evolved.

## 1. Introduction

Pathogens are disease-causing biological agents which exert some of the strongest selective pressures on animals, including humans [1–3]. The continuous evolutionary arms race between hosts and pathogens produces not only a multitude of specialized pathogens, but also complex and adaptable immune systems. Once pathogens enter the body, they are combated by the immune system. However, infections and immune responses incur high costs for the host, for example, energetic and metabolic demands, body mass loss, reduced activity and reduced reproductive success [4]. To reduce the risks and costs of exposure and infections, many animal species have evolved a broad range of behavioural adaptations [5–8]. This defence system against disease has been named the *behavioural* immune system [9,10].

In humans, the behavioural immune system is highly adaptable and results from a complex interplay between genetic predisposition, individual learning and cultural transmission [11,12]. It includes psychological mechanisms, such as detecting pathogen cues, experiencing disgust, engaging in avoidance behaviours and practising hygiene. The emotion of disgust is strongly associated with the activation of the behavioural immune system [9]. While disgust is a human emotion with universal triggers (e.g. faeces, rotten food and decaying corpses), some triggers and disgust sensitivity can vary considerably across individuals and cultures [13–15]. It has been suggested that disgust evolved as an adaptive response to protect the body from disease [13] by motivating avoidance of pathogen exposure across a broad range of cues, including contaminated food, bodily products, decay, parasites and infected individuals [16–22], but see [23]. While the psychological study of disgust has advanced significantly in recent decades, the phylogenetic origins of this disease-avoidance motivation remain less understood. If disgust initially evolved to avoid pathogens, examining pathogen avoidance behaviours in nonhuman primates can provide insights into its evolution by highlighting homologies, exaptations and analogies to human disgust.

Pathogens and parasites pose significant threats to group-living primates. In response to these selective pressures, primates have likely evolved a diverse array of cognitive and behavioural mechanisms to reduce the risk of infection. Hygienic behaviours such as grooming, varying sleeping locations or cleaning nests constitute behavioural pathogen avoidance [24]. However, engaging in pathogen avoidance behaviours can be costly for primates, as these behaviours may lead to forgoing food resources, information, protection against predators and social interactions with conspecifics [25]. Pathogen sensitivity refers to the variation in pathogen avoidance behaviours across and within individuals, groups and species. Pathogen avoidance can encompass several strategies, such as avoiding diseased conspecifics, habitats and contaminated substrates [25]. It has been theorized that parasite and pathogen stress affected the evolution of social systems in many primate species, for example, leading to subgrouping and network modularity in larger groups to reduce the spread of infections [26,27]. Social dynamics play an important role in modulating immune responses in both humans and nonhuman primates. The biological immune system is affected by individuals' social status, such as socioeconomic status in humans and dominance rank in nonhuman primates [28]. One study demonstrated that experimentally manipulating the dominance rank of female rhesus macaques (*Macaca mulatta*) causally affected their immune responses, suggesting adaptations against specific disease risks associated with specific positions within dominance hierarchies [29].

Several observational studies have documented behaviours in nonhuman primates that may reduce exposure to contaminants, including food washing and handling practices [30,31], as well as avoidance of parasitized conspecifics [32]. In addition, experimental approaches deliberately tested mechanisms, including triggers, of pathogen avoidance behaviours in primates. For instance, several studies tested food-related pathogen avoidance behaviours in primates by presenting food alongside contamination cues. Sarabian & MacIntosh [33] first introduced a paradigm in which subjects are presented with substrates with contamination cues and neutral substrates, all of which have food placed on top. Presented with both a higher and lower quality food reward (peanuts and wheat grains), wild Japanese macaques (*Macaca fuscata*) chose the higher quality food reward regardless of the substrate. However, they did show pathogen avoidance for the lower quality food reward, suggesting that the decision to feed off a contaminated substrate was contingent on the perceived food value. This indicates that food-related pathogen avoidance behaviours are trade-offs between perceived pathogenic threats and perceived caloric benefits. Subsequent studies used similar paradigms to test other primate species, including chimpanzees (*Pan troglodytes*) [34], bonobos (*Pan paniscus*) [35], mandrills (*Mandrillus sphinx*) [36], woolly monkeys (*Lagothrix lagothricha*) [37] and grey mouse lemurs (*Microcebus murinus*) [38]. Both individual (e.g. sex, age, rank, reproductive status and health) and environmental factors (e.g. season, food abundance, sick conspecifics and environmental contamination cues) can influence pathogen avoidance behaviours [25]. Taken together, results suggest that primate pathogen avoidance is influenced by ecological, social and individual factors. For example, studies found variability in pathogen sensitivity across sexes, with females showing higher pathogen avoidance than males [32,33,37,38]. However, plasticity in pathogen sensitivity in response to early life experiences or infectious disease exposure remains poorly understood. Early developmental experiences, including rearing conditions, have been documented to produce long-lasting effects on the biological immune system of macaques. For example, one study documented that rhesus macaques raised without their mothers had reduced cellular immunity and increased gastrointestinal infections compared to mother-raised individuals [39]. Additionally, ecological factors such as recent exposure to disease outbreaks may further shape pathogen sensitivity, but this has not been empirically investigated. This research gap limits our knowledge of the phylogeny of disgust and the behavioural immune system in humans and closely related species.

The genus *Macaca* is commonly used as a model for studying human immunology [40] and is also well suited for studying the evolution of pathogen avoidance. Macaques live in large multi-male, multi-female groups [41], are broadly omnivorous, and occupy a diverse range of habitats [42–44], exposing them to a wide array of pathogens and parasites, especially populations living near humans [45–47]. Due to these ecological and social characteristics, macaques share several selective pressures with humans that might be less prevalent in nonhuman great apes. Their varied environments and social structures can thus provide insights into the flexibility and evolution of the behavioural immune system.

We conducted two studies to investigate the plasticity of pathogen sensitivity, that is, the variability of responses to contamination cues, in macaques, examining the influence of early life experiences (Study 1) and exposure to a recent infectious disease outbreak (Study 2). Previous work documented that juvenile macaques observe their mothers and older conspecifics during foraging, which influences their feeding choices [48]. This emphasizes the role of social learning in macaques' dietary choices, including food-related avoidance behaviours. Further, rearing conditions have been documented to produce long-lasting effects on the biological immune system of macaques [39]. Additionally, reduced pathogen avoidance has been reported in woolly monkeys as a function of the time they spent in captivity [37]. Together, these findings suggest that species-typical social environments during early development may play a formative role in the acquisition of pathogen avoidance behaviours. We predicted that sanctuary-born macaques, raised by conspecifics, show greater pathogen avoidance than former pets, raised by humans (Study 1).

To our knowledge, no previous study has directly examined whether experiencing an infectious disease outbreak heightens pathogen avoidance behaviour in nonhuman primates. While a growing body of research documents pathogen avoidance across a wide range of animal taxa, the extent to which individuals avoid disease-related cues varies considerably across species, social systems and ecological contexts [24,25]. In highly social species living in stable, close-contact groups, such as banded mongooses (*Mungos mungo*), avoidance of diseased conspecifics may be constrained because reducing social contact may neither meaningfully reduce pathogen exposure nor outweigh the benefits of continued social interactions [8,49]. In humans, however, perceived infection risk during disease outbreaks can increase general disgust sensitivity, including responses to contamination cues unrelated to the specific disease threat [50–52]. If a similar generalized recalibration of pathogen sensitivity occurs in nonhuman primates, individuals that recently witnessed a deadly disease outbreak might show heightened avoidance of contamination cues broadly, regardless of whether those cues match the specific transmission route of the experienced disease outbreak. We predict that individuals from groups that experienced an infectious disease outbreak show heightened pathogen avoidance compared to an unexposed group (Study 2). By testing plasticity and variability in pathogen sensitivity in an Old World monkey, we aim to advance understanding of the proximate and ultimate mechanisms shaping the behavioural immune system.

## 2. Study 1: The influence of rearing history

In Study 1, we tested how food-related pathogen sensitivity is influenced by early life experiences in sanctuary-living macaques from multiple species and rearing backgrounds. Rearing backgrounds in captive primates can vary from offspring raised by their own mothers, such as sanctuary-born macaques, to offspring raised by humans, such as pet primates. We tested 54 macaques from seven different species and four different rearing backgrounds (sanctuary-reared, zoo-reared, laboratory-reared and former pets) using a sliding-table setup that presented them with two different substrates that were baited with a food reward (see figure 1). Each trial featured one *contaminated* substrate (epoxy-coated macaque faeces) and one *non-contaminated* substrate that was either neutral (piece of wood) or novel (epoxy-coated brightly coloured kitchen sponge). Each subject participated in up to eight trials, and the substrate location and alternative substrate type were counterbalanced.

**Figure 1.**
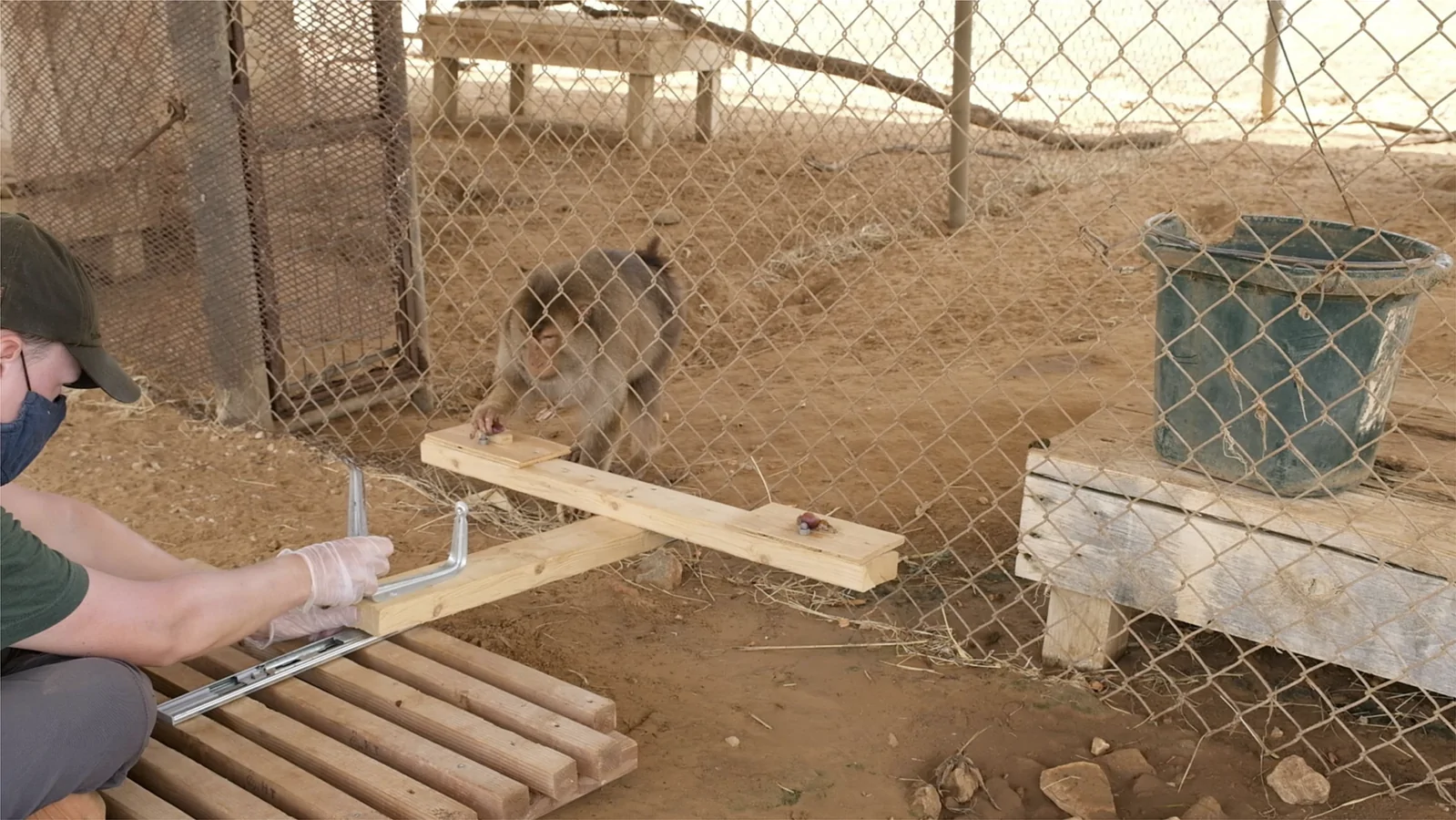
Photograph of a trial with the sliding table extended within reach of the subject. The non-contaminated neutral substrate (wood) is on the left and the contaminated substrate (faeces) is on the right. The subject, a pig-tailed macaque, chose the grape placed on top of the neutral substrate.

**Figure 2.**
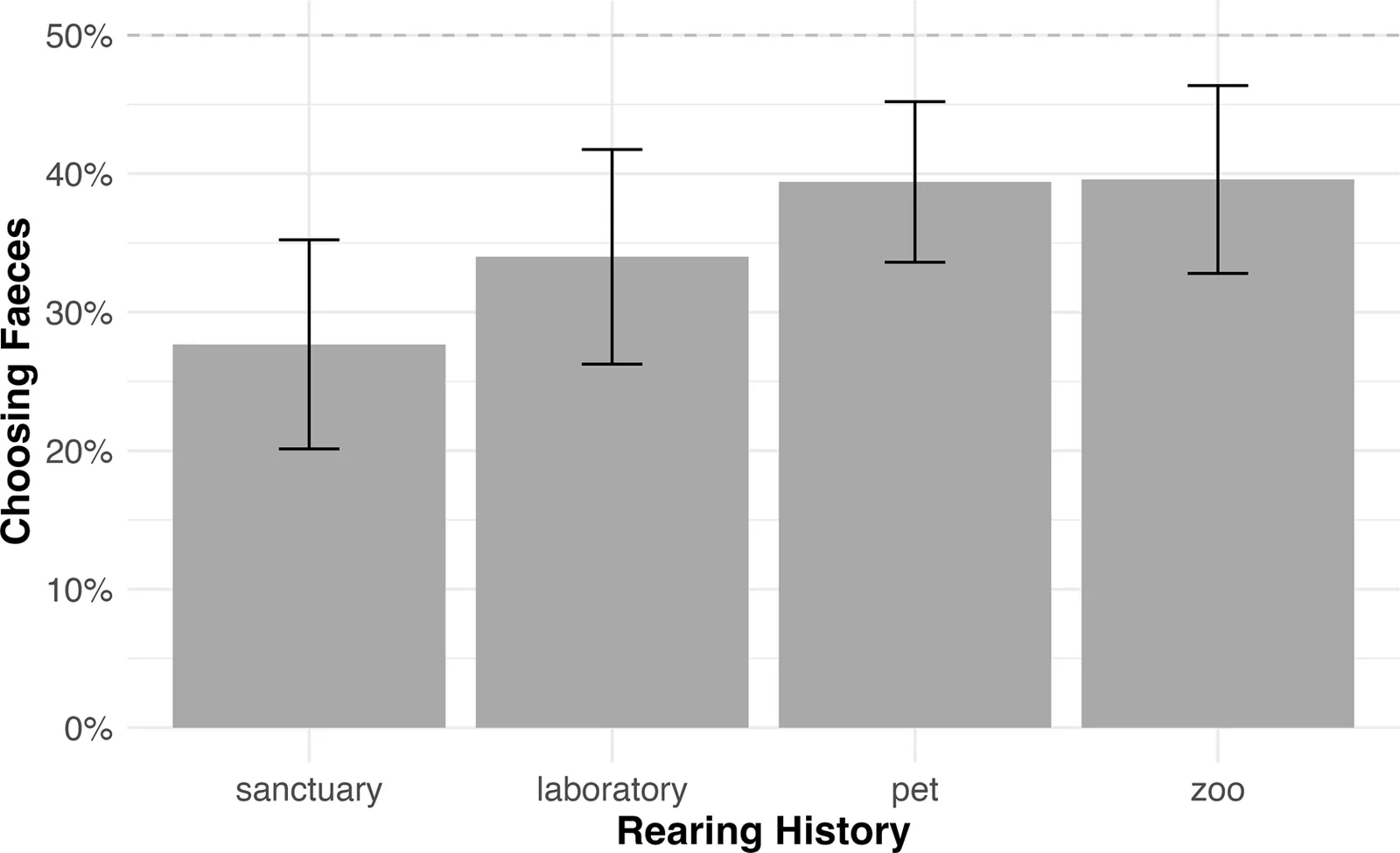
Mean percentages of subjects choosing food off the contaminated substrate (faeces) across different rearing backgrounds. Mean percentages were derived from subjects' mean choices across trials to account for inter-individual variability. Error bars represent the standard error of the mean. The grey dashed line indicates chance-level.

**Figure 3.**
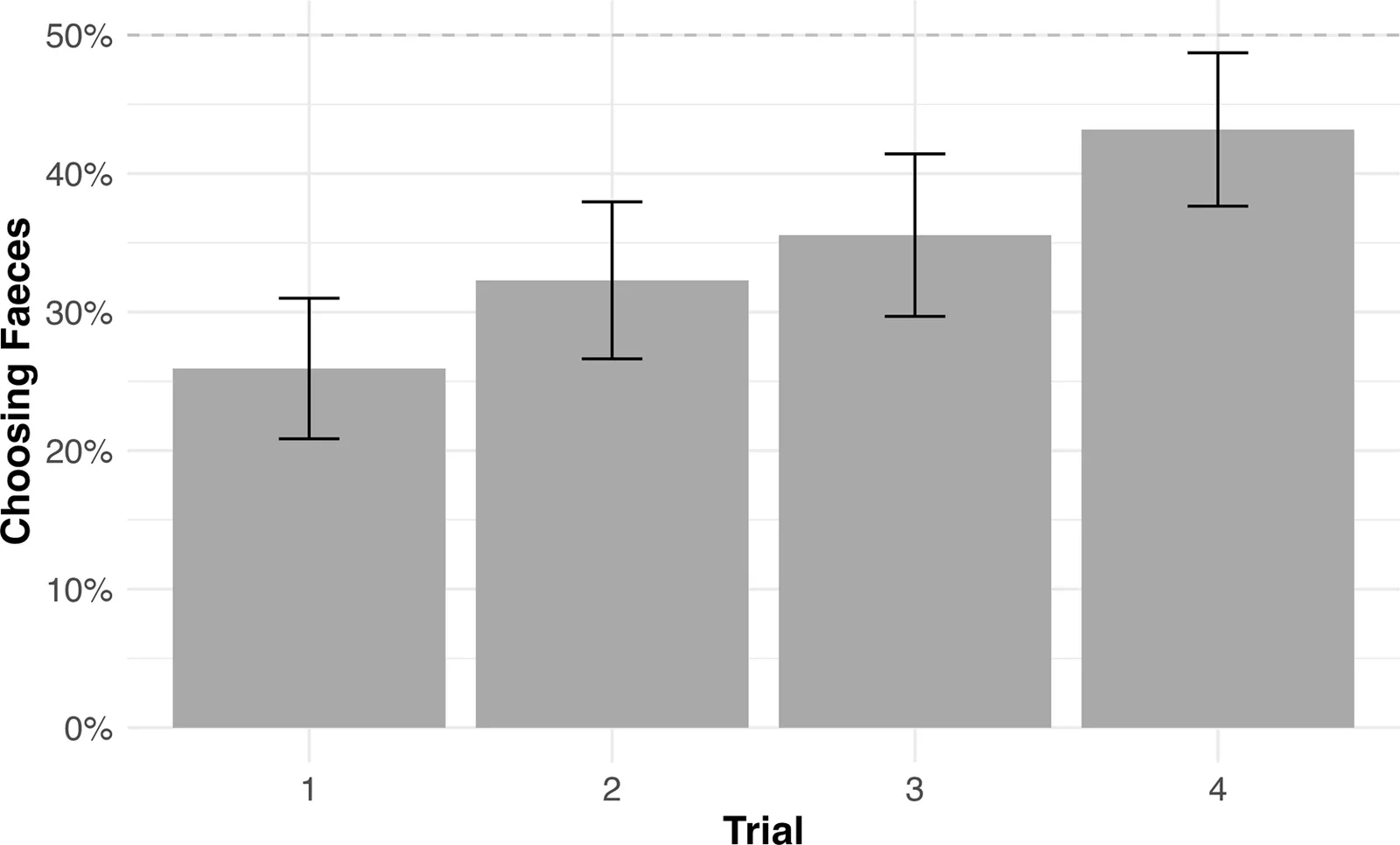
Mean percentages of subjects choosing food off the contaminated substrate (faeces) across a session. Subjects participated in up to two sessions, with four trials per alternative substrate (wood/sponge). Mean percentages were derived from subjects' mean choices across trials to account for inter-individual variability. Error bars represent the standard error of the mean. The grey dashed line indicates chance-level.

### 2.1. Study 1 results

We replicated previous findings, showing that macaques in our experiment were significantly more likely to feed on the neutral substrates compared to the contaminated substrate (intercept-only model: OR = 0.46, *p* < 0.001). Overall, subjects chose the reward on the contaminated substrate in 32% of trials (101/317). In trials using the neutral non-contaminated substrate (wood), subjects chose the reward from the contaminated substrate in 30% of trials (55/182). When confronted with a choice between the contaminated substrate (faeces) and the novel non-contaminated substrate (sponge), subjects chose to feed off the contaminated substrate in 34% of trials (46/135). We tested 52 subjects in the wood condition and 36 subjects in the sponge condition. On average, subjects completed 5.87 trials (s.d. = 2.63), with 3.5 (s.d. = 1.04) using the wood and 3.75 (s.d. = 0.77) using the sponge non-contaminated substrate.

The full model was significantly better at predicting the chosen substrate than the null model ($\chi^{2}$(7) = 14.15, *p* = 0.049). Rearing history was a significant predictor: sanctuary-born macaques were significantly less likely to choose the contaminated substrate than former pets (OR = 2.09, *p* = 0.030; see figure 2 and table 1). The contrast with zoo-reared individuals pointed in the same direction but did not reach conventional significance (OR = 2.17, *p* = 0.069), and sanctuary-born macaques did not significantly differ from former laboratory animals. Trial number was a significant predictor, with subjects becoming more likely to choose the contaminated substrate as the number of trials increased (see figure 3). Contrary to some previous studies, we did not find sex differences in our subjects. Further, rank was not a significant factor. The non-contaminated substrate condition (wood/sponge) yielded no significant differences, suggesting that pathogen sensitivity was not explained by neophobia.

**Table 1.** Model 1: predictors of substrate choice including rearing background.

predictor	odds ratio	CI	*p* value
(intercept)	0.12	[0.05, 0.31]	< 0.01^***^
background			
pet versus sanctuary	2.09	[1.07, 4.07]	0.03^*^
laboratory versus sanctuary	1.54	[0.76, 3.10]	0.23
zoo versus sanctuary	2.17	[0.94, 5.00]	0.07
sex	1.31	[0.73, 2.34]	0.37
rank	1.40	[0.81, 2.40]	0.23
trial	1.32	[1.06, 1.64]	0.01^*^
alternative substrate	0.83	[0.51, 1.37]	0.47

^*^p < 0.05, ^**^p < 0.01 and ^***^p < 0.001.

Given that the rearing background and species showed collinearity, we ran a second model, replacing the fixed effect of rearing background with species. The second model did not perform significantly better than the null model ($\chi^{2}$(10) = 11.27, *p* > 0.05), and species was not a significant predictor (see table 2).

**Table 2.** Model 2: predictors of substrate choice including species.

Predictor	odds ratio	CI	*p* value
(intercept)	0.20	[0.07, 0.55]	< 0.01^***^
species			
rhesus versus longtail	1.19	[0.53, 2.66]	0.67
bonnet versus longtail	1.40	[0.58, 3.40]	0.45
hybrid versus longtail	0.64	[0.25, 1.64]	0.35
pigtail versus longtail	0.85	[0.26, 2.77]	0.79
stumptail versus longtail	0.94	[0.43, 2.04]	0.87
Japanese versus longtail	0.95	[0.27, 3.29]	0.94
sex	1.18	[0.64, 2.17]	0.6
rank	1.59	[0.88, 2.87]	0.12
trial	1.33	[1.07, 1.65]	0.01^*^
alternative substrate	0.80	[0.49, 1.32]	0.38

^*^p < 0.05, ^**^p < 0.01 and ^***^p < 0.001.

## 3. Study 2: The influence of disease outbreaks

While our first study highlights how early life experiences shape pathogen sensitivity in adult macaques, the question remains regarding how flexible this sensitivity is in response to environmental threats such as infectious disease outbreaks. To explore macaques' adaptive flexibility in pathogen sensitivity, our second study opportunistically examines Japanese macaques' avoidance behaviours in relation to an infectious disease outbreak at the sanctuary. Using this rare opportunity of a natural experiment, we used a modified design of Study 1 to test whether the disease-exposed macaque groups showed increased pathogen avoidance compared to a non-exposed control group. We tested 55 Japanese macaques from four different groups housed in separate fenced enclosures. Three of the four groups recently experienced an infectious disease outbreak, Simian Retrovirus (SRV) Type II, which resulted in the deaths of multiple individuals. We used the same *contaminated* (macaque faeces) and *non-contaminated* (piece of wood or kitchen sponge) substrates as in Study 1. For each trial, food-baited substrates were directly placed inside each group's enclosure (see figure 4), and we coded the order and frequencies of food taken from each substrate. We analysed two measures in this study: [1] which food piece was taken first, and [2] whether the contaminated piece was eventually eaten.

**Figure 4.**
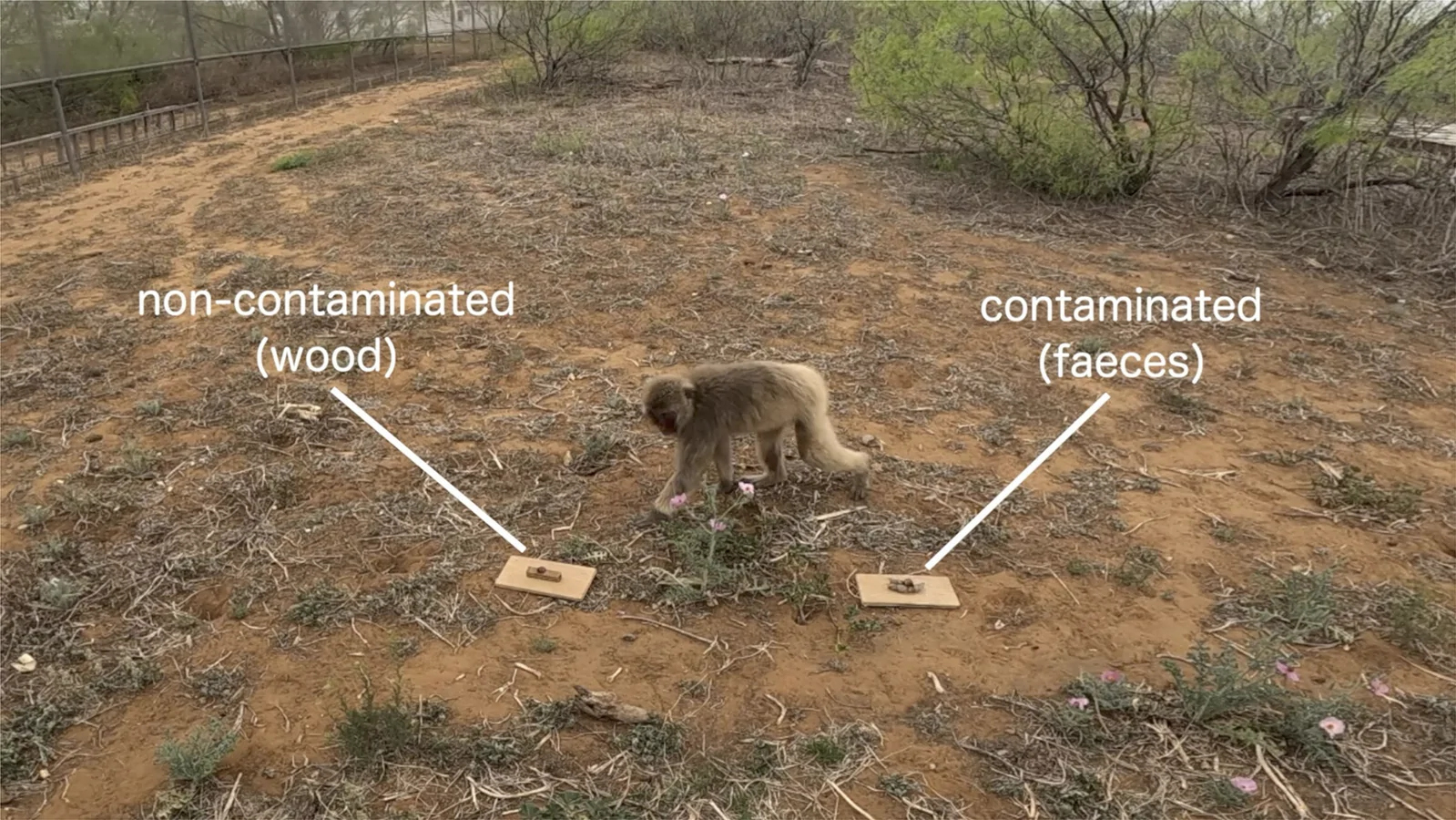
Photograph of a trial (study 2) inside a Japanese macaque enclosure (Northwest). The non-contaminated neutral substrate (wood) is on the left and the contaminated substrate (faeces) on the right side.

### 3.1. Study 2 results

We collected 281 trials (Kyoto: *n* = 103, Northwest: *n* = 76, Mirai: *n* = 52, Koshima: *n* = 50), out of which 197 (70%) were successful (Kyoto: *n* = 80, Northwest: *n* = 52, Mirai: *n* = 30, Koshima: *n* = 35). We collected at least one trial from around 60% of individuals across the groups (Kyoto: *n* = 25 [53%]; Northwest: *n* = 15 [58%]; Koshima: *n* = 11 [55%]; Mirai: *n* = 4 [27%]; see supplementary material, table S2, for subject details). On average, each subject participated in 3.6 trials (s.d. = 3.9).

Macaques were significantly more likely to feed off a non-contaminated substrate first. Indeed, they chose the contaminated substrate first in only 29% (58/197) of trials (intercept-only model: OR = 4.05, *p* < 0.001). Respectively, exposed groups chose the contaminated substrate first in 25% (29/117) of trials and the non-exposed group in 36% (29/80) of trials. In trials with the neutral non-contaminated substrate, that is, the piece of wood, macaques across all groups overall chose the contaminated one first in 22% (28/127). In trials with the novel non-contaminated substrate (sponge), they chose the contaminated one first in 43% of trials (30/70).

**Figure 5.**
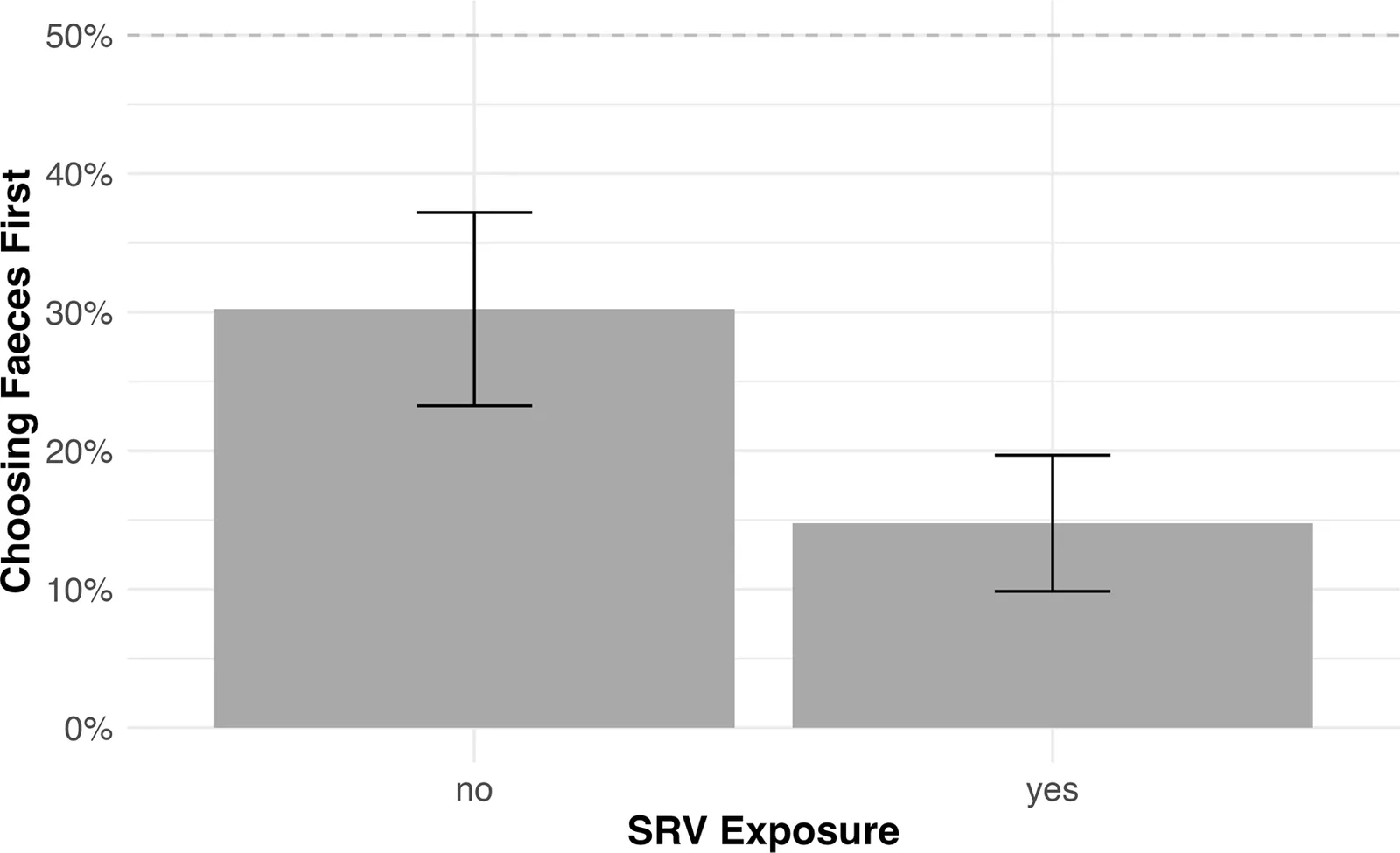
Mean percentages of subjects choosing food off the contaminated substrate (faeces) first by Simian Retrovirus (SRV) Type II exposure. Mean percentages were derived from subjects' mean choices across trials to account for inter-individual variability. Error bars represent the standard error of the mean. The grey dashed line indicates chance-level.

Model 3 performed significantly better than the null model ($\chi^{2}$(6) = 18.06, *p* = 0.006). While subjects significantly more often chose to feed off either non-contaminated substrate first, they were significantly more likely to feed off the wood substrate compared to the sponge substrate (OR = 3.42, *p* = 0.004; see table 3). The preference for feeding off the wooden substrate over the sponge substrate thus suggests a conflict between neophobia and pathogen avoidance in some subjects. With an increasing number of trials, there was a trend for subjects to become more likely to choose the contaminated substrate first, although this did not reach significance (OR = 1.10, *p* = 0.097). There was no significant difference in the likelihood of choosing to eat off the contaminated substrate first between macaques from groups that were exposed to the infectious disease and macaques from groups that were not (see figure 5). We found no significant sex difference. Rank did not affect macaques' likelihood of choosing the contaminated substrate first or eating off it. However, a minority of trials (5%) were collected from low-ranking macaques, with middle (46%) and high (49%) ranked individuals completing most trials.

**Table 3.** Model 3: predictors of first substrate choice.

Predictor	odds ratio	CI	*p* value
(intercept)	0.89	[0.22, 3.55]	0.86
disease exposure	0.37	[0.12, 1.13]	0.08
alternative substrate	0.29	[0.13, 0.67]	< 0.01^**^
sex	1.05	[0.34, 3.27]	0.93
rank			
low versus high	0.54	[0.06, 4.97]	0.59
middle versus high	0.56	[0.18, 1.77]	0.32
trial	1.10	[0.98, 1.24]	0.10

^*^p < 0.05, ^**^p < 0.01 and ^***^p < 0.001.

During each trial, subjects were able to eat both grapes. Critically, monkeys from exposed groups were far less likely to ever eat the contaminated food during a trial (47% of trials; 55/117) than monkeys from the unexposed control group (79% of trials; 63/80). Therefore, monkeys from exposed groups were far more likely to refuse the food placed on contaminated substrates entirely and only ate the piece off the non-contaminated substrate, whereas monkeys from non-exposed groups typically ate both pieces.

**Figure 6.**
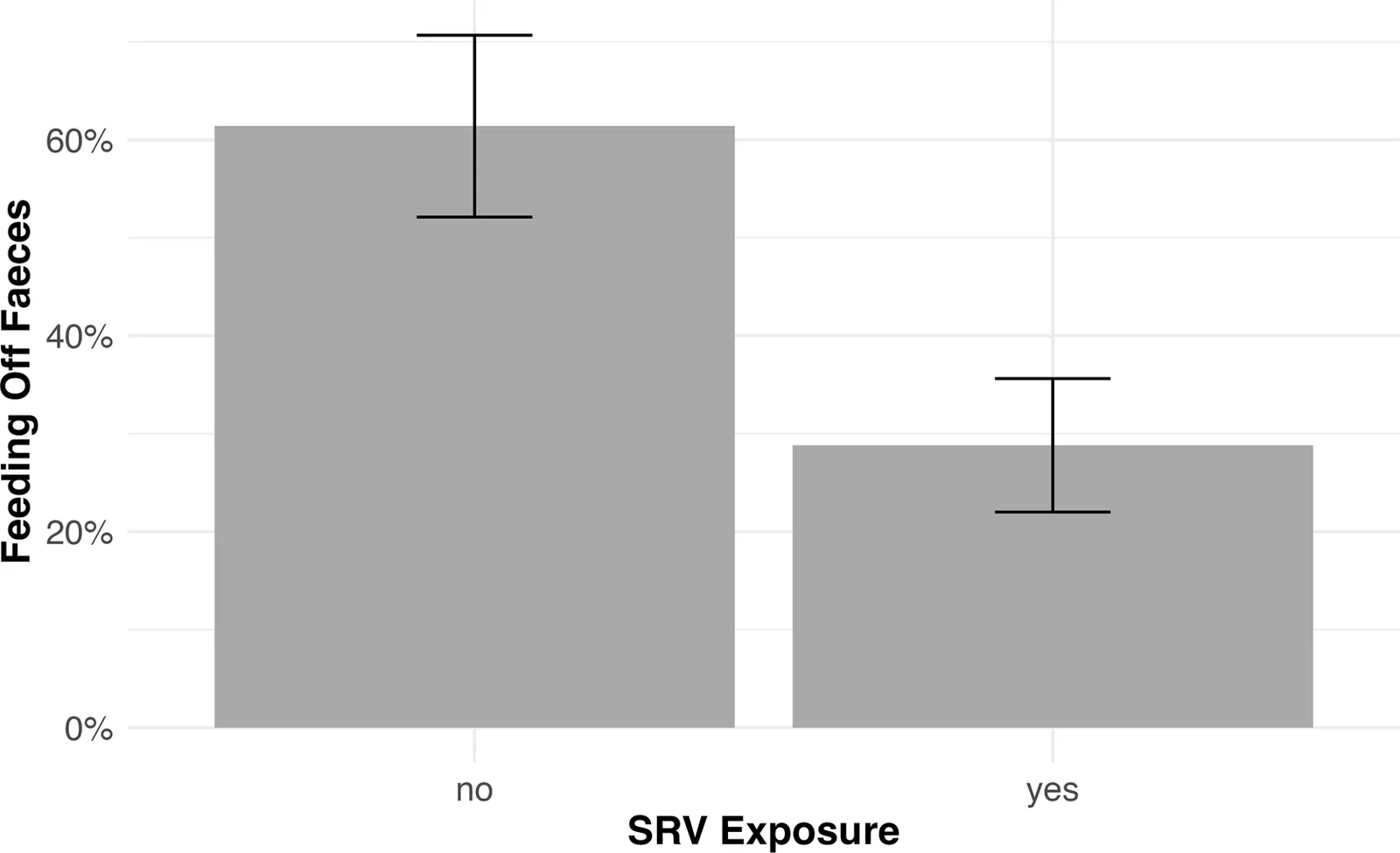
Mean percentages of subjects choosing food off the contaminated substrate (faeces) within a trial by Simian Retrovirus (SRV) Type II exposure. Mean percentages were derived from subjects' mean choices across trials to account for inter-individual variability. Bars show the mean of those subject-level percentages and error bars represent the standard error of the mean across subjects.

Model 4 performed significantly better than the null model ($\chi^{2}$(6) = 24.38, *p* < 0.001). Subjects from groups exposed to the disease were significantly less likely to feed off the contaminated substrate (OR = 0.07, *p* = 0.007; see figure 6 and table 4). Their likelihood of feeding off the contaminated substrate increased across trials (OR = 1.27, *p* = 0.02). The non-contaminated substrate type did significantly influence their likelihood of feeding off the contaminated substrate (OR = 0.22, *p* = 0.01). There was no significant difference between the sexes.

**Table 4.** Model 4: predictors of feeding off faeces.

predictor	odds ratio	CI	*p* value
(intercept)	8.54	[0.85, 85.48]	0.07
disease exposure	0.07	[0.01, 0.47]	< 0.01^**^
alternative substrate	0.22	[0.07, 0.73]	0.01^*^
sex	1.53	[0.26, 9.00]	0.64
rank			
low versus high	0.27	[0.01, 5.55]	0.40
middle versus high	0.57	[0.09, 3.53]	0.55
trial	1.27	[1.03, 1.56]	0.02^*^

^*^ *p* < 0.05, ^**^p < 0.01 and ^***^p < 0.001.

For an exploratory examination of whether the group-level effect of outbreak exposure was carried by having experienced the outbreak or by being infected, we additionally assigned each individual an SRV infection status (see Methods). Excluding the six Northwest individuals who were never tested left 49 individuals and 173 trials. Individuals from outbreak-exposed groups fed from the contaminated substrate on a similar proportion of trials whether or not they had themselves been infected (uninfected: 33.2%, SEM 11.9, *n* = 9; infected: 31.7%, SEM 10.5, *n* = 15), and both differed from the unexposed group (61.4%, SEM 9.3, *n* = 25). In a model containing both predictors, outbreak exposure remained a significant predictor of feeding off the contaminated substrate (OR = 0.05, *p* = 0.016) whereas individual infection status did not (OR = 2.92, *p* = 0.394). Adding exposure to a model already containing infection status improved model fit ($\chi^{2}$(1) = 6.38, *p* = 0.012), whereas adding infection status to a model already containing exposure did not ($\chi^{2}$(1) = 0.69, *p* = 0.405). The same pattern held for first choice, where neither predictor was significant (see supplementary material, table S3).

## 4. Discussion

We investigated food-related pathogen avoidance in sanctuary-living macaques across two studies. In both experiments, subjects displayed a strong preference for feeding from non-contaminated substrates over faecally contaminated ones. Importantly, avoidance behaviour and thus pathogen sensitivity varied systematically with early life experience and with recent exposure to infectious disease. We found significant differences at both the individual level (based on the macaque's rearing background) and the group level (based on whether the group had experienced a recent infectious disease outbreak). These results indicate that the capacity for a flexible, environmentally responsive behavioural immune system is not unique to humans but is also present in Old World monkeys.

In our first study, we demonstrated pathogen avoidance across multiple macaque species, extending previous findings to species not examined before (bonnet, pig-tailed, stump-tailed, rhesus and pig-tailed–long-tailed hybrid macaques). Notably, we found that rearing history was the strongest predictor of pathogen sensitivity in our tested population. Sanctuary-born macaques raised by their conspecific mothers showed significantly greater avoidance of contaminated food than former pet macaques raised by humans. This suggests that a species-typical environment during early development, one with opportunities to observe and learn hygienic behaviours from conspecifics, plays a role in the development of pathogen avoidance. By contrast, individuals lacking such early exposure to conspecifics, that is, former pets raised by humans, showed reduced avoidance responses. Zoo-born macaques in our sample also tended toward lower pathogen sensitivity, but differences involving this group were not statistically significant, likely due to small sample sizes and diverse rearing backgrounds. Notably, sanctuary-born macaques did not differ significantly from former laboratory animals in their avoidance of contaminated substrates. Macaques bred at research facilities in the United States are generally reared by their biological mothers in social groups [53], meaning that laboratory-reared individuals likely had more early exposure to conspecifics than former pets. It is also possible that the relevant variable is not rearing history per se but rather the duration of time spent in captivity or the age at which individuals arrived at the sanctuary. Reduced pathogen avoidance as a function of years in captivity has been reported in woolly monkeys [37]. Due to incomplete records, we could not reconstruct the detailed biographies of macaques prior to their arrival at the sanctuary. This limitation should be considered when interpreting the rearing-background effects. Future studies with detailed life-history records could help to assess the relative influences of early rearing environment and cumulative captivity experience. Within our study, we did not find any differences across species. These might be either absent or too difficult to disentangle from rearing effects. In practice, species differences within macaques might be less influential than the influence of early life history.

Our second study demonstrated that pathogen sensitivity can rapidly increase following acute infectious disease threats. Sanctuary-born Japanese macaques from groups that had recently experienced a deadly infectious disease outbreak showed markedly higher avoidance of the contaminated substrate compared to macaques from a non-exposed control group. In other words, individuals in the outbreak-exposed groups were more willing to forgo a food reward than to ingest food that made contact with a potentially contaminated surface. This heightened aversion to contamination in the aftermath of an outbreak might reflect an adaptive recalibration of the behavioural immune system in response to more perceived pathogenic threat, for example in the form of diseased or deceased conspecifics. We interpret our results as evidence of group-level flexibility in avoidance behaviour in the face of elevated disease risk.

An important question is what mechanism underlies the heightened avoidance in disease-exposed groups. One possibility is that the experience of witnessing sick and dying conspecifics during the outbreak led to a generalized increase in pathogen sensitivity, that is, a recalibration of the behavioural immune system in response to elevated perceived disease risk. An alternative, and not mutually exclusive possibility is that individuals with compromised immune systems due to SRV infection behaviourally compensated by increasing avoidance of potential pathogen sources. Because infection and the immune challenge that accompanies it can themselves alter immune function, exposure to an outbreak may in principle recalibrate both defence systems, the biological immune system as well as the behavioural one, and the two forms of recalibration are not mutually exclusive. Notably, SRV is transmitted primarily through blood and saliva via bite and scratch wounds, and faecal–oral transmission has not been documented [54–56]. The heightened avoidance of faecally contaminated substrates in exposed groups can therefore not be straightforwardly explained by learned avoidance of the specific transmission route. Instead, we interpret this as more consistent with a generalized upregulation of pathogen sensitivity. Our main analysis defines SRV exposure at the group level, that is, whether or not a group experienced the outbreak, irrespective of the infection status of individual subjects. At the time of testing, the health status of SRV-positive individuals varied: some showed no clinical symptoms, while others had developed chronic immunodeficiency. To separate these two accounts, we compared group-level outbreak exposure with individual infection status directly (see Study 2 Results and supplementary material, table S3). Individuals from outbreak-exposed groups behaved similarly whether or not they had themselves been infected, and both differed from the unexposed group. The behavioural shift therefore tracks having experienced the outbreak rather than having been infected by it, which is more consistent with the behavioural account than with the immunological one. However, we treat this comparison as exploratory because only nine outbreak-exposed individuals are confirmed uninfected. The confidence interval for infection status is wide enough to remain compatible with a substantial effect, and only one enclosure contained both infected and uninfected individuals, so infection status is only partly separable from group.

Infectious disease events can drive lasting changes in primate behaviour. A well-known case in wild baboons illustrates how a disease outbreak can alter social dynamics: a tuberculosis outbreak that killed the most aggressive males led to a long-term shift toward a more peaceful group culture [57]. In our study, although the outbreak did not drastically change group membership, it coincided with a clear increase in avoidance behaviour in the affected groups. It remains uncertain whether this behavioural shift arose through selection of more avoidant individuals, individual learning or social learning. Primates can develop group-specific feeding behaviours through social learning [58,59], raising the possibility that the heightened avoidance in our tested macaque groups was socially transmitted. Future observations should examine how long the elevated avoidance persists and whether new group members adopt these behaviours, which would indicate cultural transmission of this pathogen-avoidance strategy rather than individual changes and learning.

Consistent with previous observations, we found evidence of habituation to the contamination stimulus over repeated trials in Study 1 [36]. With successive trials within a session, monkeys became more willing to take food from the contaminated substrate, suggesting they became desensitized to the sight of our visually contaminated substrate, which lacked smell and touch contamination cues. A comparable waning of faeces avoidance across repeated presentations has been reported in other primates tested with visual contamination cues [36]. Trial number also predicted feeding from the contaminated substrate in Study 2, but we interpret that effect more cautiously because trial number is cumulative across the whole study rather than a position within a session, and individuals differed considerably in how many trials they contributed. We therefore restrict the habituation interpretation to Study 1. We also observed that avoidance was driven by pathogen cues rather than by novelty. In Study 1, subjects were just as willing to eat a reward off a familiar clean surface as from a novel clean surface, indicating that an unfamiliar substrate alone did not deter them. In Study 2, monkeys showed a significantly lower likelihood to feed off the novel than the familiar non-contaminated substrate, which could be due to the more limited exposure to man-made objects in the Japanese macaque population compared to our population in Study 1. Nonetheless, the presence of contamination cues was a stronger feeding deterrent than any neophobic response: in both studies, aversion to the contaminated food option consistently exceeded reluctance to feed on novel items.

We found no significant sex differences in pathogen avoidance in either study. Some previous studies in nonhuman primates documented greater pathogen avoidance in females [33,37,38,60], which has been attributed to the demands of maternal care and pregnancy, although these explanations were not tested directly. In our sample, however, none of the females was pregnant or caring for infants, which may have reduced any flexible female-specific tendency for heightened pathogen sensitivity. Another possibility is that sex differences observed elsewhere reflect underlying risk-taking tendencies rather than sex itself. In humans, individuals with higher disgust sensitivity tend to engage in fewer risky behaviours [61–63]. If females generally take fewer risks, they may in turn be more pathogen-avoidant. Future studies could explore the relationship between pathogen avoidance and risk-taking in primates and consider reproductive status when examining potential sex effects.

Several limitations of our studies should be noted. First, Study 2 uses a between-subjects design. We have no measure of avoidance for these individuals before the outbreak. Therefore, we cannot exclude stable individual differences as a contributor to the group differences we report. Lacking pre-outbreak baseline data in Study 2, we cannot rule out alternative explanations to the outbreak causally leading to group-level differences in pathogen sensitivity. For example, groups might have differed in avoidance even before the disease event, with our control group showing unusually low pathogen sensitivity. Additionally, selective mortality during the outbreak could have influenced the results: if less-avoidant individuals were more likely to die, the post-outbreak group compositions could consist of more avoidant individuals. We consider this scenario unlikely, since the disease spread mainly via biting rather than via contaminated food. Therefore, infection risk was likely tied more to involvement in aggressive interactions than variation in hygienic tendencies. Moreover, many monkeys in the affected groups survived infection asymptomatically, suggesting survival did not depend solely on hygienic behaviours. In Study 1, incomplete information about some subjects' early-life conditions (especially for zoo-born and ex-pet individuals) introduced variability that, along with small sample sizes, may have masked subtle effects, for example of species differences. Future studies with baseline measurements and detailed life-history data would help confirm the causes of the patterns we observed.

Our findings have practical implications for primate management and conservation. The significant influence of early life history on pathogen avoidance highlights the importance of species-typical social environments in captivity. For example, ensuring that young primates are raised by their mothers or in rich conspecific social groups (rather than by humans) could help them develop appropriate pathogen sensitivity and avoidance. This is especially relevant for populations intended to be released back into wild environments, where recognizing and avoiding pathogen cues could improve survival.

Regarding the evolution of disgust, our findings suggest that flexible, context-sensitive pathogen avoidance is present in Old World monkeys and shaped by both developmental and ecological experience. Disgust in humans likely did not arise *de novo* but was built upon an older system of behavioural disease avoidance whose sensitivity is calibrated by phylogenetic and ontogenetic history. While our studies did not measure disgust directly, our results demonstrate the kind of experience-dependent plasticity in pathogen sensitivity that, in humans, is a hallmark of the disgust system [11,22].

In sum, our findings support the presence of a flexible behavioural immune system in macaques, who last shared a common ancestor with humans approximately 25 to 30 million years ago. The capacity to modulate pathogen sensitivity and avoidance behaviours according to environmental pathogen risks in an Old World monkey raises the question of whether such flexibility is homologous or analogous to humans. If this plasticity is widespread across Old World monkeys, it likely represents a homologous trait; if it is confined mainly to species with high pathogenic risks (e.g. large-group, terrestrial and omnivorous taxa), convergent evolution could be a more likely explanation. To that end, examining pathogen sensitivity in species with different social systems and ecologies, such as highly arboreal, pair-living gibbons or semi-solitary orangutans, could be particularly informative.

## 5. Methods

### 5.1. Study 1

#### 5.1.1. Subjects

We tested macaques (*n* = 54) from seven different species, including one hybrid species: *M. arctoides* (*n* = 9); *M. fascicularis* (*n* = 14), *M. fuscata* (*n* = 3), *M. mulatta* (*n* = 11), *M. nemestrina* (*n* = 3); *M. radiata* (*n* = 8) and *M. fascicularis x nemestrina* (*n* = 6). These macaques were socially housed at the Born Free USA Primate Sanctuary in Texas. Testing was conducted within the macaques' everyday enclosures, consisting of indoor and outdoor spaces with natural vegetation and enrichment (see supplementary material, table S1, for subject details). We physically separated subjects from group members whenever possible by testing them within smaller lockable compartments within their enclosures. Only subjects accustomed to temporary separation were tested in isolation; others were tested with enclosure mates present. Testing did not interfere with the animals' daily feeding schedule, and all subjects had *ad libitum* access to water throughout all data collection. All animals were fed daily and received similar amounts of food, minimizing the possibility of variation in food abundance influencing pathogen sensitivity. Information on the sex, age and rearing background of each subject was obtained from the sanctuary's internal database. Our study was approved by the Institutional Animal Care and Use Committee (IACUC) of the University of California, San Diego (S21059). All materials and procedures were approved by the sanctuary team, which included a veterinarian and professional animal caretakers.

#### 5.1.2. Apparatus

We built a wooden sliding table (width: 80 cm, length: 100 cm and height: 15 cm) to present the macaques with food in two distinct locations (left/right), approximately 60 centimetres apart. Each location allowed for the attachment of one substrate on top of which food rewards could be placed. The sliding table consisted of a base plate and a T-shaped top plate that was attached with a drawer slide, allowing the food locations to slide approximately 35 centimetres. Three substrates of comparable sizes and shapes were used (see supplementary material, figure S1): a piece of wood (non-contaminated neutral), a kitchen sponge piece (non-contaminated novel), and macaque faeces (contaminated). Each substrate was fixed on a rectangular piece of plywood (12 × 20 cm) and secured to the sliding table using nuts and bolts.

The wood substrate was familiar to the monkeys, as their enclosures included structures built from similar plywood, thereby functioning as a neutral control substrate. Macaque faeces for the contaminated substrate were collected from healthy Japanese macaques housed within the sanctuary. The faeces were covered in a layer of transparent epoxy plastic, creating a visual contamination cue without olfactory or tactile contamination cues. Previous research suggested that visual pathogen cues were sufficient to trigger food-related pathogen avoidance behaviours in macaques [33]. The epoxy gave the contamination substrate a wet appearance, resembling freshly excreted faeces. We decided to only use visual contamination cues to standardize our stimuli across trials, minimize the risk of any infections for subjects, and ensure that substrates would not taint the taste of the food reward to avoid measuring distaste. We also coated a bright blue- and yellow-coloured piece of kitchen sponge with epoxy, creating a second non-contaminated substrate, which served as a control for both neophobia and the appearance of epoxy coating on the contaminated substrate. Substrates were attached to a rectangular plywood plate, which could be secured to either side of the sliding table using nuts and screws.

#### 5.1.3. Procedure

We presented the macaques with a food-choice task using the substrates and sliding table, which was set outside the enclosure fence for each subject. Fully extending the table rendered both substrates within the subject's reach. For each trial, two identical pieces of food were placed on two of the substrates. We used red grape halves for the majority of trials, and banana slices were used occasionally for a small number of individuals when grapes were not available. Peanuts were used as rewards for long-tailed macaques in accordance with the sanctuary's nutrition and veterinary guidelines, given that some were prediabetic and diabetic.

An occluder constructed with cardboard was placed over the apparatus during the setup of each trial to minimize the subjects' exposure to the substrates. A trial began once the apparatus was baited, the occluder was removed, and the sliding table was extended within reach of the subject. The apparatus was retracted by the experimenter once a piece of food was taken to prevent the subject from accessing the other food reward. This setup ensured that subjects could only retrieve food from one substrate per trial, limiting their caloric intake per session and preventing conspecifics from accessing the other food item for sessions during which subjects could not be separated.

Each trial lasted until one piece of food was taken off a substrate or until four minutes passed without the subject making a choice. If the subject did not take a piece of food within 2 minutes, the experimenter threw an additional piece of food between the two substrates inside the enclosure to attract them to the setup. A trial was repeated if the apparatus malfunctioned or the subject timed out due to a lack of choice within the 4 minutes. Testing for an individual subject was stopped for the day if they had two subsequent timeouts. A maximum of eight trials per subject were collected, run in two blocks of four trials each, where the contaminated substrate was paired with either the neutral substrate (wood) or the novel substrate (sponge). The order of the non-contaminated substrates was counterbalanced across subjects, and the location (left/right) of the contaminated substrate was pseudorandomized for each trial block. This resulted in six different randomizations with the contaminated substrate placed two trials on the left and two trials on the right. Data collection took place between March 2022 and March 2024. All trials were video-recorded using either a *GoPro Hero 11* or a *Fujifilm XT-4,* placed between approximately 2 and 4 metres away from the sliding table. Examples of trials are shown in supplementary material, video S1.

#### 5.1.4. Coding and statistical analysis

We scored each video recorded, coding the identity of the subject, chosen side, and chosen substrate. To assess inter-rater reliability, a second coder (A.G.) coded 20% (*n* = 63) of all trials. Cohen's kappa was calculated for chosen side and chosen substrate using the ‘kappa2’ function of the ‘irr’ package in R [64]. The agreement between raters was perfect for the chosen side (*κ* = 1) and chosen substrate (*κ* = 1) [65].

We asked caretakers to assess rank for each subject based on dominance (low/middle/high). We categorized subjects into four different rearing backgrounds based on animal records, namely sanctuary-born individuals (*N* = 14), former pets (*N* = 15), former laboratory research animals (*N* = 19), and zoo-born animals (*N* = 6; see supplementary material, table S1). Macaques that were categorized as sanctuary-raised were born at the Born Free USA Primate Sanctuary or the Wild Animal Orphanage from San Antonio, which relocated their macaques to Born Free USA after shutting down in 2010. Sanctuary-born macaques were raised by their biological mothers. Former pets are usually raised by humans under species-inappropriate conditions, which was confirmed for all former pets whose history was documented in the sanctuary's internal database. The exact rearing histories of former laboratory animals could not be reconstructed; however, macaques used in invasive research in the United States are generally reared by their biological mothers [53]. The exact rearing history of former zoo animals could not be reconstructed from the database. Zoo-born primates are usually raised by their biological mothers; however, maternal rejection and health concerns oftentimes lead to rearing by human caretakers.

All data analyses were performed in R, version 4.5.1 [66]. We ran two binomial generalized linear mixed models (GLMMs) using the ‘glmer’ function of the ‘lme4’ package [67]. Both models assess the predictors of choosing to feed off the contaminated substrate. The two models share the same structure and differ only in whether rearing background or species is included, because the two are collinear (see Results). Model 1 included rearing background (sanctuary/zoo/laboratory/pet), sex (m/f), dominance rank (high/low), trial number and condition (wood/sponge) as fixed effects; Model 2 replaced rearing background with species. Trial number in Study 1 refers to the position of a trial within a four-trial substrate session (1–4), not to a cumulative count. To test the effect of rearing background, sanctuary-born was used as the reference level. The reported contrasts thus reflect comparisons of each rearing category against sanctuary-born individuals, in line with our prediction that sanctuary-born individuals show higher pathogen avoidance than the other backgrounds. The identity of the subject was included as a random effect with a random intercept. We compared each full model to a corresponding null model (containing only the intercept and subject identity as a random effect) using likelihood ratio tests (LRT) via the ‘anova’ function in R with a chi-square test statistic.

### 5.2. Study 2

#### 5.2.1. Subjects

We tested Japanese macaques (*n* = 55) from four different groups (Koshima, Kyoto, Mirai and Northwest) housed at the Born Free USA Primate Sanctuary. All animals from these groups are the descendants of the Arashiyama West troop, a group from the Arashiyama population that was transplanted from Japan to Southern Texas in 1972 [68]. The groups live in separate fenced enclosures with natural vegetation characteristic of the Tamaulipan mezquital, a semi-arid brushland featuring vegetation such as mesquites, thorny shrubs, acacias and cacti. The enclosures range between 0.6 and 2 hectares in size. The macaques are fed daily with a balanced diet consisting of fresh produce, fruits, sweet potatoes, nuts and monkey chow. Food is distributed across several spots in the enclosure once a day between 8 am and 11 am, minimizing direct clumped food competition within the groups. All animals had *ad libitum* access to water through several water troughs with drinking spouts inside their enclosures.

In January 2020, Born Free USA confirmed the presence of Simian Retrovirus (SRV) Type II in their Japanese macaque population. SRV-II is transmitted primarily through blood and saliva, typically via bite and scratch wounds sustained during aggressive interactions [54–56]. Faecal-oral transmission has not been described as a route of infection for SRV. Infected individuals often suffer from acute, deadly progressions that lead to severe haemorrhage. During non-acute progressions, individuals may continue to carry the virus in a latent phase and appear non-symptomatic. However, the disease can progress chronically, leading to immunodeficiency. Progressive weight loss and increased susceptibility to infections and other diseases can occur. SRV shares similarities with AIDS in humans, which is caused by HIV.

A thorough investigation established that the disease was brought into the sanctuary by a non-symptomatic long-tailed macaque, who was taken in after retiring from biomedical laboratory research. Around the last quarter of 2019, the disease likely mutated and was transmitted to a Japanese macaque, eventually spreading through some groups of the Arashiyama population. SRV-II has rarely been documented in Japanese macaques but is described as one of the most pathogenic types. The sanctuary immediately increased its already existing stringent hygiene and quarantining protocols, successfully limiting the spread of the disease to other enclosures.

Some infected individuals passed away quickly from severe haemorrhage, while others developed long-term autoimmune diseases. To date, the further spread of the disease into other groups has been successfully prevented. The sanctuary also implemented a strict testing and quarantining protocol to separate SRV-negative monkeys from positive ones. Positive-testing individuals from the *Main* enclosure were moved into a new enclosure and are now called the *Koshima* group. Negative-testing individuals from *Main* were moved into a separate enclosure and are now called the *Mirai* group. Therefore, animals from three of the four groups in our study (Northwest, Koshima and Mirai) were exposed to the disease outbreak, with the non-exposed group (Kyoto) functioning as a control group.

We asked the lead caretaker at the sanctuary to assign ranks (high rank, middle rank and low rank) to each monkey to get an estimate of their dominance status. One group (Koshima) also included one male long-tailed macaque (Reggie), whose trials (*N* = 3) were included in the analyses since he lived with the Japanese macaques before the SRV outbreak. However, we also analysed the data excluding his trials, which did not change the results of any model.

#### 5.2.2. Procedure

We used the same stimuli as in Study 1. However, instead of using a sliding table, we placed both substrates directly on the ground within each group's enclosure and moved them out of view from the setup trial. Therefore, we did not control which monkey would engage in any given trial. This was necessary, given that the monkeys were not habituated to the presence of researchers. After baiting both substrates, the experimenter moved away from the setup. Two substrates at a time (faeces-wood and faeces-sponge) were placed approximately one metre apart from each other on the ground. Each trial lasted around 10 minutes to leave enough time for a monkey to discover the setup, but simultaneously limiting the exposure and habituation of monkeys to the study materials. We varied the placement location across each trial, choosing spots that were not frequented by monkeys during setup. The aim was to give as many different subjects a chance to discover the baited substrates as possible. In this setup, it was possible for the subjects to take only one or both grape halves. We only counted trials where a subject arrived at the setup with both grapes still on the substrates. All trials were video recorded using *GoPro Hero 7* or *GoPro Hero 11* action cameras, which were placed between approximately 3 and 6 metres away from the substrates. Trials were collected across three different field trips ranging from October 2022 to March 2024. Examples of trials are shown in supplementary material, video S2.

#### 5.2.3. Coding and statistical analysis

We scored each video-recorded trial by coding the identity of the subject, their first choice and whether they made a second choice. Again, a second coder (A.G.) coded 20% (*n* = 40) of all trials to assess inter-rater reliability using Cohen's *κ*. The agreement for the first (*κ* = 0.96) and second choice (*κ* = 0.96) was almost perfect. A trial was considered successful if at least one food reward was taken off one of the substrates by a macaque. While it sometimes occurred that the second reward was taken off by a second individual, we only analysed second choices that were made by the same individual that took the first reward. We categorized animals from the enclosures Northwest, Koshima and Mirai as exposed to the infectious disease, whereas animals from Kyoto were categorized as non-exposed. SRV exposure was defined as groups experiencing the infectious disease outbreak, irrespective of the infection status of any specific individual. For an additional analysis, we also assigned each individual an SRV infection status from the sanctuary's veterinary PCR and serology records. All individuals in Kyoto and Mirai were uninfected, all individuals in Koshima were infected (apart from Reggie, who tested negative on both tests), and in Northwest, where only part of the group was tested, individual test results were used. Individuals that were never tested were coded as unknown and excluded from that analysis instead of being assigned a presumed status. The status of each individual is given in the deposited dataset. We ran two binomial generalized linear mixed models using the same methods as specified in the first study. The first model predicts the first choice (contaminated/non-contaminated). Given that subjects were able to pick both food rewards, we ran a second model to assess predictors for feeding off faeces within a trial. Both models include exposure to the infectious disease (yes/no), sex (male/female), dominance rank (high/middle/low), condition (wood/sponge) and trial number as fixed effects. Trial number in Study 2 is cumulative per individual across the whole study period. We included individual identity as a random effect with a random intercept. Again, each full model was compared to a corresponding null model (containing only the intercept and subject identity as a random effect) using likelihood ratio tests (LRT) via the ‘anova’ function in R with a chi-square test statistic. For the exploratory analysis of infection status, we refitted Model 4 on the 173 trials from the 49 individuals whose SRV status was known, entering group-level SRV exposure (yes/no) and individual infection status (positive/negative) as separate fixed effects alongside the same covariates and the same random intercept. To assess whether each term accounted for variance beyond the other, we fitted three models: one containing exposure only, one containing infection status only, and one containing both. We compared these by AIC and, since each single-predictor model is nested within the model containing both, by likelihood ratio tests using the same procedure as above. We then repeated the model containing both predictors for the first choice.

## Supplementary Material



## Data Availability

All data and code used for this study are are available on Dryad [69]. Supplementary material is available online [70].
