# Supplementary material for: Early-life experience and disease exposure affect pathogen avoidance behaviour in macaques
Source: R Soc Open Sci. 2026 Oct 21;13(10):260011. doi: 10.1098/rsos.260011 (PMC13692775; doi:10.1098/rsos.260011)
Supplement: Supplementary file 1 [file rsos.260011.zip › rsos.260011/rsos260011_si_003.docx]

**Supplementary Information for**
Early-life Experience and Disease Exposure Affect Pathogen Avoidance Behaviour in Macaques

**Table S1.** Study 1 subjects’ name, species, sex, date of birth (DoB), enclosure and rearing background.

| **Species** | **Name** | **Sex** | **DoB** | **Enclosure** | **Background** |
| --- | --- | --- | --- | --- | --- |
| *M. arctoides* | Abo | male | 2002-11-29 | WAO-3 | sanctuary |
| *M. arctoides* | Brenda | female | 1995-02-12 | WAO-2 | laboratory |
| *M. arctoides* | Courtney | female | 1995-08-16 | Trustees | zoo (HVZ) |
| *M. arctoides* | Dewey | male | 2011-03-06 | WAO-3 | sanctuary |
| *M. arctoides* | Fatima | female | 1996-11-29 | Trustees | zoo (HVZ) |
| *M. arctoides* | Lycan | male | 2007-05-22 | WAO-3 | sanctuary |
| *M. arctoides* | Malcolm | male | 2007-09-05 | WAO-3 | sanctuary |
| *M. arctoides* | Stewey | male | 1992-01-03 | WAO-3 | zoo (HVZ) |
| *M. arctoides* | Trixie | female | 1993-11-27 | Trustees | zoo (HVZ) |
| *M. fascicularis* | Benny | male | 2004-04-01 | WAO-7 | laboratory |
| *M. fascicularis* | Billy | male | 1990-06-01 | WAO-4 | laboratory |
| *M. fascicularis* | Chuckie | male | 2005-07-01 | WAO-LT | laboratory |
| *M. fascicularis* | Ernie | male | 2005-07-01 | WAO-LT | laboratory |
| *M. fascicularis* | Felix | male | 1995-01-01 | KhyTown | laboratory |
| *M. fascicularis* | Freeman | male | 2001-11-18 | Bab1 | pet |
| *M. fascicularis* | Frick | male | 1996-10-13 | KhyTown | laboratory |
| *M. fascicularis* | Gilbert | male | 1998-07-10 | Meadows2D | pet |
| *M. fascicularis* | Gizmo | male | 2004-07-01 | WAO-1 | laboratory |
| *M. fascicularis* | Mo | male | 2006-08-19 | WAO-4 | sanctuary |
| *M. fascicularis* | Mose | female | 2001-08-26 | WAO-4 | sanctuary |
| *M. fascicularis* | Sissy | female | 2002-01-01 | Bab1 | pet |
| *M. fascicularis* | Tiny | male | 2004-07-01 | WAO-1 | laboratory |
| *M. fascicularis* | Whitey | male | 2005-07-01 | WAO-LT | laboratory |
| *M. fuscata* | Khy | male | 2007-07-02 | Gilligan’s | pet |
| *M. fuscata* | Lucy | female | 2011-05-23 | Haven1B | sanctuary |
| *M. fuscata* | Ollie | male | 1998-06-01 | Gilligan’s | pet |
| *M. mulatta* | Audrey | female | 2003-01-20 | Meadows1A | pet |
| *M. mulatta* | Colleen | female | 1995-04-28 | WAO-5 | zoo |
| *M. mulatta* | Creed | male | 2005-01-11 | Haven2B | laboratory |
| *M. mulatta* | Eddie | male | 1994-01-01 | Meadows2D | zoo |
| *M. mulatta* | Gambit | male | 2016-07-30 | Meadows2A | pet |
| *M. mulatta* | Lyla | female | 2016-02-24 | Meadows1B | pet |
| *M. mulatta* | Maude | female | 1995-01-01 | Haven1A | laboratory |
| *M. mulatta* | Oliver | male | 2008-05-28 | Haven1A | pet |
| *M. mulatta* | Oscar | male | 2004-10-14 | Haven2B | laboratory |
| *M. mulatta* | Professor | male | 2000-07-14 | Meadows1A | laboratory |
| *M. mulatta* | Quinn | male | 2000-05-01 | WAO-5 | sanctuary |
| *M. nemestrina* | India | female | 2002-01-01 | Meadows2D | pet |
| *M. nemestrina* | Nala | female | 1997-11-27 | WAO-8 | pet |
| *M. nemestrina* | Tazman | male | 1996-05-01 | Meadows2A | pet |
| *M. radiata* | Armstrong | male | 2003-05-07 | WAO-6 | laboratory |
| *M. radiata* | Bernie | male | 2009-06-16 | WAO-6 | pet |
| *M. radiata* | Clarice | female | 2004-11-03 | WAO-6 | laboratory |
| *M. radiata* | Grinch | male | 1997-01-16 | WAO-6 | laboratory |
| *M. radiata* | LeMond | male | 2003-09-09 | WAO-6 | laboratory |
| *M. radiata* | LilBit | female | 2005-07-24 | WAO-1 | laboratory |
| *M. radiata* | Max | male | 2011-04-07 | WAO-6 | pet |
| *M. radiata* | Poppy | male | 2010-04-14 | WAO-6 | pet |
| *M. fa. x ne.* | Connie | female | 2008-09-06 | Meadows2A | sanctuary |
| *M. fa. x ne.* | Mungojerry | male | 2011-07-13 | WAO-4 | sanctuary |
| *M. fa. x ne.* | Nadine | female | 2012-08-04 | WAO-8 | sanctuary |
| *M. fa. x ne.* | Nigel | male | 2011-06-13 | WAO-8 | sanctuary |
| *M. fa. x ne.* | Pushkin | male | 2006-08-19 | WAO-4 | sanctuary |
| *M. fa. x ne.* | Talbot | male | 2010-07-31 | WAO-4 | sanctuary |

**Table S2.** Study 2 subjects’ name, sex, (estimated) date of birth, SRV exposure, and enclosure.

| **Name** | **Sex** | **DoB** | **SRV Exposure** | **Enclosure** |
| --- | --- | --- | --- | --- |
| Arcadia | female | 2000-01-01 | yes | Koshima |
| Barry | male | 2004-01-01 | yes | Koshima |
| Brock | male | 2001-01-01 | yes | Koshima |
| Daphne | female | 2003-11-05 | yes | Koshima |
| Ginger | female | 2003-11-05 | yes | Koshima |
| Jackyboy | male | 2010-05-01 | yes | Koshima |
| Jolie | female | 2005-06-01 | yes | Koshima |
| Joy | female | 2003-01-01 | yes | Koshima |
| Kate | female | 1998-05-01 | yes | Koshima |
| Kyle | male | 2004-01-01 | yes | Koshima |
| Larry | male | 2012-05-01 | yes | Koshima |
| Mabuchi | male | 2002-01-01 | yes | Koshima |
| Reggie | male | 2000-05-01 | yes | Koshima |
| Ally | female | 2002-11-06 | no | Kyoto |
| Amanzi | female | 2002-01-01 | no | Kyoto |
| Banzai | male | 2017-06-01 | no | Kyoto |
| Boyfriend | male | 2002-01-01 | no | Kyoto |
| Cal | male | 2016-06-01 | no | Kyoto |
| CrazyEyes | female | 2002-01-01 | no | Kyoto |
| Dandy | female | 2011-11-03 | no | Kyoto |
| Eric | male | 2016-06-01 | no | Kyoto |
| Ethel | female | 2002-01-01 | no | Kyoto |
| Finn | male | 1999-01-01 | no | Kyoto |
| Hector | male | 2011-05-01 | no | Kyoto |
| Jameson | male | 2000-05-01 | no | Kyoto |
| Junichi | male | 2000-01-01 | no | Kyoto |
| Kat | male | 2001-01-01 | no | Kyoto |
| Maly | female | 2002-11-06 | no | Kyoto |
| Marx | male | 2012-05-01 | no | Kyoto |
| Maureen | female | 2022-11-06 | no | Kyoto |
| Milly | female | 1995-01-01 | no | Kyoto |
| Mustachio | female | 2002-01-01 | no | Kyoto |
| OrphanAnnie | female | 2016-06-01 | no | Kyoto |
| Pranny | male | 2012-05-01 | no | Kyoto |
| Sebastian | male | 1999-01-01 | no | Kyoto |
| Summer | female | 1995-01-01 | no | Kyoto |
| Tilly | female | 2004-01-01 | no | Kyoto |
| Trey | male | 2004-01-01 | no | Kyoto |
| Tumnus | male | 2010-05-01 | no | Kyoto |
| Venus | female | 2013-11-03 | no | Kyoto |
| Angelina | female | 1998-01-01 | yes | Mirai |
| Herman | male | 2003-01-01 | yes | Mirai |
| Kimmy | female | 2005-01-01 | yes | Mirai |
| Ryan | male | 2002-01-01 | yes | Mirai |
| Albus | male | 2000-01-01 | yes | Northwest |
| Arthur | male | 2003-01-01 | yes | Northwest |
| Chester | male | 2000-01-01 | yes | Northwest |
| Cleo | female | 1998-01-01 | yes | Northwest |
| Cooper | female | 2002-01-01 | yes | Northwest |
| Erin | female | 2005-01-01 | yes | Northwest |
| Gordan | male | 2002-01-01 | yes | Northwest |
| Heather | female | 1998-01-01 | yes | Northwest |
| Jo | male | 2000-01-01 | yes | Northwest |
| Kazumi | female | 2003-01-01 | yes | Northwest |
| Komachi | female | 2000-01-01 | yes | Northwest |
| Marge | female | 2001-01-01 | yes | Northwest |
| Masanobu | male | 2005-01-01 | yes | Northwest |
| Mindy | female | 2002-01-01 | yes | Northwest |
| Parker | male | 2005-01-01 | yes | Northwest |
| Tang | female | 2002-01-01 | yes | Northwest |
| Tippy | female | 2000-01-01 | yes | Northwest |

**Table S3.** Comparison of group-level outbreak exposure with individual SRV infection status as predictors of feeding off the contaminated substrate in Study 2. Individuals whose infection status is unknown, i.e. the six Northwest animals that were never tested, are excluded, leaving 49 individuals and 173 trials. Because the Northwest group contains both infected and uninfected individuals, the two predictors are not perfectly confounded and can be entered in the same model. All models include the alternative substrate, sex, dominance rank and cumulative trial number as covariates and a by-subject random intercept, as in Model 4.

**(a) Subject-level descriptive statistics**

| **Group** | **Individuals** | **Trials** | **Ate faeces, subject-level mean %** | **SEM** |
| --- | --- | --- | --- | --- |
| Unexposed, uninfected | 25 | 80 | 61.4 | 9.3 |
| Exposed, uninfected | 9 | 45 | 33.2 | 11.9 |
| Exposed, infected | 15 | 48 | 31.7 | 10.5 |

**(b) Model comparison**

| **Model** | **AIC** | **Exposure OR [95% CI], p** | **Infection OR [95% CI], p** |
| --- | --- | --- | --- |
| Outbreak exposure only | 163.84 | 0.08 [0.010, 0.68], p = 0.020 | - |
| Individual infection status only | 169.53 | - | 0.31 [0.030, 3.32], p = 0.335 |
| Both predictors | 165.15 | 0.05 [0.004, 0.57], p = 0.016 | 2.92 [0.249, 34.34], p = 0.394 |
| Both predictors (first choice) | 209.48 | 0.49 [0.134, 1.77], p = 0.276 | 1.22 [0.280, 5.35], p = 0.788 |

**(c) Likelihood-ratio tests**

| **Comparison** | **Likelihood-ratio test** |
| --- | --- |
| Adding exposure to a model already containing infection status | chi2(1) = 6.38, p = 0.012 |
| Adding infection status to a model already containing exposure | chi2(1) = 0.69, p = 0.405 |

**Figure S1. Photograph of the three different substrate types used in study 1.** From left to right: wood (non-contaminated neutral), epoxy-coated sponge (non-contaminated novel), epoxy-coated faeces (contaminated). Additional substrates of each type were built for study 2.

**Video S1.** Three example trials from Study 1.

**Video S2.** Three example trials from Study 2.
